# Mature mediastinal teratoma with somatic type malignancy including neuroblastoma and intestinal type of adenocarcinoma: A Case Report

**DOI:** 10.1016/j.ijscr.2021.105680

**Published:** 2021-02-26

**Authors:** Maciej Rachwalik, Kinga Kosiorowska, Maciej Bochenek, Marek Jasinski, Roman Przybylski

**Affiliations:** Department of Cardiac Surgery, Wroclaw Medical University, Wroclaw, Poland

**Keywords:** Mediastinal tumour, Mediastinal teratoma, Neuroblastoma, Adenocarcinoma

## Abstract

**Introduction and importance:**

The majority of mediastinal tumours develop asymptomatically and are often detected incidentally on a chest X-ray performed for another reason. Mediastinal tumours, although mostly asymptomatic, may cause non-specific symptoms associated with advanced tumour growth.

**Case presentation:**

We present a case of a 30-year-old woman who presented with exhaustion and lower back pain accompanied by severe headaches with symptoms of visual disturbances, followed by the typical Horner syndrome. Computed tomography revealed a tumour measuring 12 × 11 × 10 cm in the right cavity with features suggestive of teratoma. The patient underwent mediastinal tumour resection and thymectomy. The pathomorphological examination confirmed the primary diagnosis of mediastinal teratoma, but rare somatic type malignancy was detected. Therefore, the patient was referred for further oncological treatment.

**Discussion:**

Mediastinal teratoma is an uncommon finding and usually asymptomatic. Despite its slow growth, it can grow enough to compress adjacent structures, causing symptoms similar to those presented in our patient.

**Conclusion:**

Radiologic imaging proves diagnostic in most cases. Despite the somatic type malignancy, surgical excision of the tumour using the en-bloc technique seems to be a sufficient option for the patient, and further oncological treatment is not always obligatory.

## Highlights

• Mediastinal tumours may cause non-specific symptoms, associated with tumour growth.
• Mediastinal teratoma is uncommon finding and most often present in young adults.
• Total surgical excision is the treatment of choice for mediastinal teratoma.
• Imaging study appears to be a sufficient tool to diagnose mediastinal teratoma.

## 1 Introduction

The majority of mediastinal tumours develop asymptomatically and are often detected incidentally on a chest X-ray performed for another reason [1]. The most common aetiologies include thymomas, lymphomas, germ cell tumours, and thyroid masses. Sporadically, mediastinal tumours cause symptoms that are not specific and often associated with advanced tumour growth that puts pressure on surrounding structures. The onset of symptoms like chest pain, cough, dyspnoea, dysphagia, or respiratory failure is related to tumour advancement and is an indication, for often lifesaving, surgical intervention [2]. Written informed consent was obtained from the patient for publication of this case report and any accompanying images. This case has been reported in line with the SCARE criteria [3].

## 2 Presentation of case

**Fig. 1:**
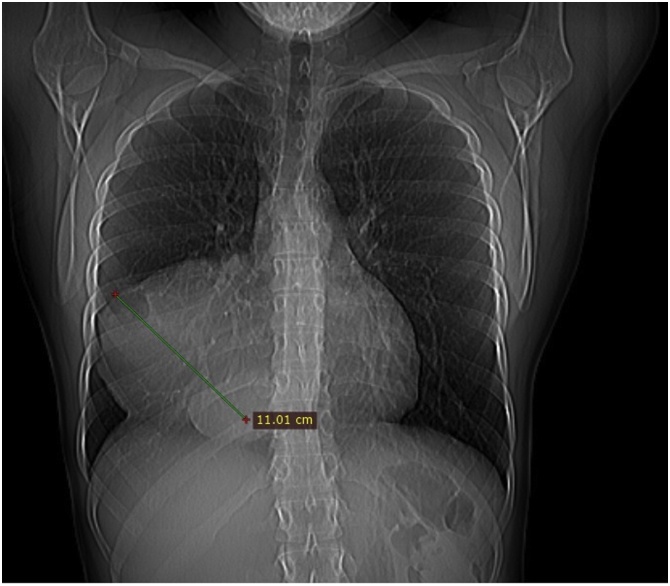
Pre-operative chest x-ray.

**Fig. 2:**
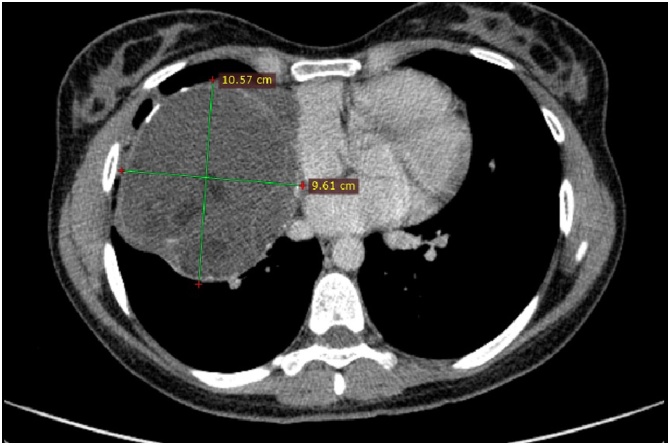
Axial contrast-enhanced CT image at the level of the pulmonary artery trunk shows a round, anterior mediastinal mass with heterogenous content.

**Fig. 3:**
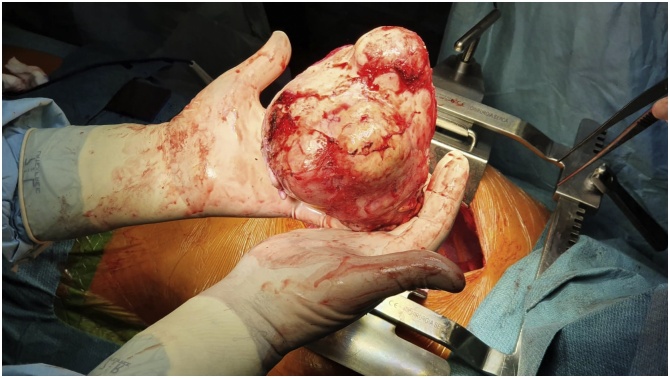
En-block tumour resected from the right thoracic cavity.

A 30-year-old female patient with an active and healthy lifestyle was feeling fine until two months when exhaustion and lower back pain began. The symptoms were initially associated with intense work effort. A week later, she developed severe headaches with symptoms of visual disturbances, photophobia, orbital pain, and a general decline in immunity. Soon, the symptoms were accompanied by the loss of skin sensation in the upper part of the forehead, an uneven pupil diameter, and a dropping right eyelid, which prompted the patient to consult a physician. At the time of medical evaluation, a chest X-ray revealed an opacity in the right lung field extending from the mediastinum, obliterating the hilar and right lower lung shadow (Fig. 1). A CT scan revealed a well-defined cystic mass in the right thoracic cavity, shifting the heart slightly to the left, entering the superior mediastinum, and reaching the jugular notch, measuring 12 × 11 × 10 cm (Fig. 2). The mass presented with a heterogeneous content, with numerous cysts, calcifications, and areas of adipose tissue, most likely corresponding to the teratoma image. The patient was discussed in the Heart Team and, with the consent of all specialists, was then advised for urgent surgery performed by cardiac surgeons. Following median sternotomy, the tumour was observed in the anterior mediastinum and was tightly adherent to the surface of the right middle and lower lung lobe, and the pericardium. The patient underwent a mediastinal tumour excision and thymectomy. The mass was attached to the pericardium, however, there was no need to proceed with pericardiectomy as the tumour did not penetrate the pericardium and was easy to dissect. There were no visible invasions of adjacent structures. The mass en-block excision and thymectomy revealed a well-defined tumour with a maximum diameter of 10 cm (Fig. 3). The postoperative course was uneventful, and the symptoms totally resolved. Histopathology revealed a mature mediastinal teratoma (60%) with somatic type malignancy (40%) including neuroblastoma and intestinal type of adenocarcinoma. There was no evidence of invasion outside the tumour capsule, and the operation was classified as R0. The patient was referred to the oncology centre for further evaluation. Positron emission tomography (PET) did not reveal any suspicious areas of enhanced metabolic activity. The most common laboratory tumour markers were all negative. At 3 months follow-up, she returned to her former physical fitness and sports activity, without any signs of disease.

## 3 Discussion

Teratoma is a rare type of germ cell tumour containing tissues derived from one or more germ layers i.e., ectoderm, mesoderm, or endoderm. The vast majority of cases are benign, and in very few cases they are prone to malignancy and metastasis. Mediastinal teratoma is an uncommon finding with an incidence of 10–15% of all mediastinal tumours, and most often present in young adults with a mean age of 20–40 years old [1,2]. It is very difficult to diagnose at an early stage. They generally grow slowly and can become enormous so that they compress adjacent structures, like in the presented case. Rarely, due to the size or the enzymes produced, the tumour can rupture and erode into the pericardium or pleural cavity [2,4]. Complete surgical excision with careful separation from the surrounding tissue is the treatment of choice for benign mediastinal teratoma [5]. The most commonly performed method is median sternotomy due to excellent exposure. We report a case of symptomatic young women who presented with anterior mediastinal teratoma. Our initial diagnosis was based on the chest CT scan, which is the imaging technique of choice in the evaluation of the abnormal mediastinum. The diagnosis was confirmed by histopathological examination of the surgically resected tumour. A further histological study revealed a malignant transformation which occurs very rarely and reports a high mortality rate [4]. Since complete resection is the recommended treatment for benign teratoma, there is no consensus as to the treatment of a malignant transformation. Some researchers suggest the implementation of chemotherapy, as an adjuvant for surgical excision [6]. As we reported, our oncologists, having reviewed the case and based on the necessary tests, ruled out residual disease and metastases. Thus, the oncological treatment was completed, and the surgery deemed definitive.

We also want to draw the reader’s attention to the presence of typical Horner syndrome in our patient. It is of greatest importance to exclude myasthenia or paraneoplastic syndrome at an early stage of the diagnostic process. Laboratory tests that include ACHR, CRP, AFP, β-hCG, and other common tumour markers seem to be crucial [7]. The reason why the patient suffered from severe headache and a general decline in immunity was unclear; this might have been due to atelectasis, or exogenous activity of the adenocarcinoma component of the tumour. The PET performed postoperatively demonstrated no definite metabolic activity areas in the brain, ruling out metastatic brain disease.

## 4 Conclusion

In conclusion, we reported a case of the mature mediastinal teratoma with somatic type malignancy including neuroblastoma and intestinal type of adenocarcinoma successfully treated with complete surgical resection. Despite the malignant component there was no need for further oncological treatment. As it was in our patient, radiologic imaging can with a high accuracy prove diagnostic. Therefore, especially in the era of COVID-19, chest X-rays should be still the basic and frequently performed screening study that may reveal chest tumours at a very early stage.

## Declaration of Competing Interest

The authors report no declaration of interest.

## Funding

None.

## Ethical approval

This is a case report study. Informed patient written consent has been obtained and all identifying information was omitted.

## Consent

Written informed consent was obtained from the patient for publication of this case report and accompanying images. A copy of the written consent is available for review by the Editor-in-Chief of this journal on request.

## Author contribution

MR: Conceptualization; Supervision; Validation; Roles/Writing - original draft; Writing - review & editing; KK: Roles/Writing - original draft; Writing - review & editing; MB: Data curation; Investigation; Writing - review & editing; MJ: Supervision; Validation; Writing - review & editing; RP: Data curation; Investigation; Methodology; Supervision; Validation.

## Registration of research studies

Not applicable.

## Guarantor

Roman Przybylski.

## Provenance and peer review

Not commissioned, externally peer-reviewed.
